# Clinical reasoning in pragmatic trial randomization: a qualitative interview study

**DOI:** 10.1186/s13063-023-07445-3

**Published:** 2023-06-27

**Authors:** Justin T. Clapp, Cassandra Dinh, Monica Hsu, Mark D. Neuman

**Affiliations:** 1 Department of Anesthesiology & Critical Care, University of Pennsylvania Perelman School of Medicine, Blockley Hall, 3rd floor, 423 Guardian Dr, PA 19104 Philadelphia, USA; 2 Center for Perioperative Outcomes Research and Transformation, University of Pennsylvania Perelman School of Medicine, Philadelphia, PA USA; 3 Leonard Davis Institute of Health Economics, University of Pennsylvania, Philadelphia, PA USA; 4 IQVIA Inc., NJ Parsippany-Troy Hills, USA; 5 Rutgers Robert Wood Johnson Medical School, Piscataway, NJ USA

**Keywords:** Clinical trial, Pragmatic trial, Clinical reasoning, Equipoise, Gatekeeping, Enrollment, Recruitment, Randomization, Learning health system, Chart-stimulated

## Abstract

**Background:**

Pragmatic trials, because they study widely used treatments in settings of routine practice, require intensive participation from clinicians who determine whether patients can be enrolled. Clinicians are often conflicted between their therapeutic obligation to patients and their willingness to enroll them in trials in which treatments are randomly determined and thus potentially suboptimal. Refusal to enroll eligible patients can hinder trial completion and damage generalizability. In order to help evaluate and mitigate clinician refusal, this qualitative study examined how clinicians reason about whether to randomize eligible patients.

**Methods:**

We performed interviews with 29 anesthesiologists who participated in REGAIN, a multicenter pragmatic randomized trial comparing spinal and general anesthesia in hip fracture. Interviews included a chart-stimulated section in which physicians described their reasoning pertaining to specific eligible patients as well as a general semi-structured section about their views on clinical research. Guided by a constructivist grounded theory approach, we analyzed data via coding, synthesized thematic patterns using focused coding, and developed an explanation using abduction.

**Results:**

Anesthesiologists perceived their main clinical function as preventing peri- and intraoperative complications. In some cases, they used prototype-based reasoning to determine whether patients with contraindications should be randomized; in others, they used probabilistic reasoning. These modes of reasoning involved different types of uncertainty. In contrast, anesthesiologists expressed confidence about anesthetic options when they accepted patients for randomization. Anesthesiologists saw themselves as having a fiduciary responsibility to patients and thus did not hesitate to communicate their inclinations, even when this complicated trial recruitment. Nevertheless, they voiced strong support for clinical research, stating that their involvement was mainly hindered by production pressure and workflow disruptions.

**Conclusions:**

Our findings suggest that prominent ways of assessing clinician decisions about trial randomization are based on questionable assumptions about clinical reasoning. Close examination of routine clinical practice, attuned to the features of clinical reasoning we reveal here, will help both in evaluating clinicians’ enrollment determinations in specific trials and in anticipating and responding to them.

**Trial registration:**

Regional Versus General Anesthesia for Promoting Independence After Hip Fracture (REGAIN). ClinicalTrials.gov NCT02507505. Prospectively registered on July 24, 2015.

**Supplementary Information:**

The online version contains supplementary material available at 10.1186/s13063-023-07445-3.

## Background

Tension between the priorities of patient care and clinical research has been a central issue in modern medicine since the rise of the clinical trial in the mid-twentieth century [1]. It initially surfaced as debate about how evidence produced by trials should be integrated into practice [2–4]. Now, in the era of the “learning health system,” [5] researchers and policymakers are focused not only on how to translate findings into practice, but increasingly on how to embed research studies into everyday clinical processes. The pragmatic clinical trial, for example, by comparing common treatments in real clinical conditions using broad eligibility criteria [6], has been posed as a means of producing data with greater efficiency and generalizability than traditional explanatory trials [7].

Because they are so thoroughly embedded in practice, pragmatic trials pique the conflict between clinicians’ therapeutic obligation to individual patients and their ability to enroll patients in trials in which treatments are randomly determined and potentially suboptimal [8]. A great deal of bioethical work has been devoted to assessing how clinicians should navigate this conflict. This analysis has revolved around the concept of equipoise: a state of uncertainty about which treatment option would be better for a patient [9, 10]. When in this state, a clinician is typically seen as justified in allowing a patient to be randomized. However, after decades of discussion on the topic, there remains substantial disagreement over how to determine whether a clinician’s stance about a given patient is ethically acceptable [10–15]. In recent years, experts in ethics and policy have argued that the threshold for equipoise should be low. For example, proponents of the learning health system contend that, due to the scarcity of research evidence, everyday clinical decisions often present substantial uncertainty, and thus, the risks presented to patients randomized into trials are not necessarily greater than those presented by normal clinical care [16–20].

Among trialists, conceptual debates about how to define equipoise tend to be viewed as esoteric [21]. Trialists are concerned about the conflict between the therapeutic obligation and trial enrollment because it can prevent physicians from entering otherwise eligible patients into studies. Low enrollment is a frequent cause of premature trial termination and associated waste of time and resources [22–24], and selective enrollment of eligible patients by participating clinicians can harm the generalizability of results by excluding patients with certain features. The trial literature rarely attempts to interrogate whether clinicians are justified in refusing to randomize patients, instead seeking ways to prevent this. Sophisticated suites of mixed-methods techniques for identifying and addressing enrollment issues in individual trials have been developed [25, 26].

Given its centrality to the problem, there has been surprisingly little work characterizing how clinicians determine whether particular patients are appropriate for trial randomization. A series of studies have examined informed consent conversations, demonstrating how participating physicians’ tendencies to issue recommendations and to use imbalanced language about trial arms can inhibit recruitment [27–32]. However, clinicians’ inferences about therapeutic options appear to have a substantial influence on whether and how they talk to patients and families about trials [27, 29–31, 33, 34]. This suggests that clinical reasoning is crucial to whether patients wind up successfully randomized. An in-depth examination of clinical reasoning might present novel ways to evaluate the appropriateness of clinicians’ actions and to effectively facilitate trial enrollment [20]. Accordingly, this qualitative interview study examined the reasoning of physicians working at sites of a multicenter pragmatic randomized trial comparing spinal versus general anesthesia for hip fracture surgery. Our goal was not to judge whether the decision to proceed with or to refuse randomizing an otherwise eligible patient represented the right or wrong determination in any given scenario. Rather, by asking participating anesthesiologists to walk us through how they approached the cases of specific eligible patients, we sought to gain detailed insight into how clinicians determined whether patients were suitable for randomization.

## Methods

### Design

In this qualitative study, we used in-depth interviewing, an approach advantageous for eliciting detailed accounts of clinical reasoning in the complex setting of a pragmatic trial. We were guided by a constructivist grounded theory approach [35], which strives to develop explanations “grounded” in the exploration of empirical data while emphasizing that the sophistication of these explanations depends also on the background knowledges of the researchers involved. Because clinical reasoning in pragmatic trials is poorly characterized, the flexible, data-immersed approach of constructivist grounded theory combined with the open-endedness of interviewing allowed us to follow the data to the topics of importance rather than restrict those topics in advance. This study is part of an increasing emphasis on the use of qualitative methods to help explain clinical trial results [36].

### Setting

Regional Versus General Anesthesia for Promoting Independence After Hip Fracture (REGAIN) was a multicenter pragmatic randomized trial evaluating spinal versus general anesthesia for hip fracture surgery in previously ambulatory adults aged 50 years or older [37]. The trial was conducted at 46 sites in the USA and Canada from 2016 to 2021. Patients were randomly assigned to either general endotracheal anesthesia with inhaled anesthetic or single-shot spinal anesthesia with sedation as needed for comfort. The primary outcome was a composite of death or an inability to walk 10 ft independently at 60 days post-randomization.

The site trial staff obtained randomization assignments from a central system and evaluated the inclusion and exclusion criteria using in-person interviewing and medical record review. Patients or proxies provided informed consent for participation in the trial. Anesthesia was then administered by the usual clinical anesthesia staff at each site. REGAIN used broad eligibility criteria to maximize generalizability. However, patients determined by the research staff to otherwise meet the eligibility criteria could be excluded if physicians considered them unsuitable for randomization based on clinical assessment. The trial staff assessed 22,022 patients for eligibility, and 1600 were ultimately enrolled. Over the course of the trial, 1328 patients were excluded due to clinician refusal.

### Interviewing

We used a purposive sampling approach. We first identified 5 REGAIN sites with relatively high numbers of enrolled patients and site lead investigators who were willing to assist us with recruiting participants. Carrying out this interview study at sites that enrolled relatively high numbers was deemed necessary to provide sufficient cases to discuss with each interviewee during the interview. For each of these sites, we compiled the cases in which patients were either successfully randomized or excluded due to anesthesiologist refusal. We sent this list to the site PI, who identified the anesthesiologist associated with each case. We then sent an email to each identified anesthesiologist informing them of the interview study and inviting them to participate. Participants provided verbal consent and were compensated $75 for participating.

Interviews were one-on-one encounters conducted via video chat or phone call. The interviewers were CD (an anthropology graduate student and REGAIN clinical research coordinator), MH (a medical student with an anthropology background), and JC (a PhD anthropologist with experience conducting qualitative studies in perioperative settings). Prior to the interview, the participant was sent the list of REGAIN cases that would be discussed and asked to review them. The interview consisted of two sections (the interview guide can be found in the Supplemental Material). The first section used chart-stimulated recall, a case-based interview approach often used to examine clinical decision-making [38]. Given the complexity of clinical reasoning, having clinician interviewees view their documentation about a patient during an interview helps to stimulate recall and enrich accounts by grounding them in concrete clinical contexts.

During the chart-stimulated portion of our interviews, we questioned anesthesiologists about 2–4 cases on which they were the anesthesiologist of record and in which either (a) the patient was successfully randomized or (b) the patient, though meeting REGAIN’s eligibility criteria, was excluded from the study by the clinical team. The number of cases we reviewed per interviewee and the specific mix of randomized versus excluded cases varied depending on the frequency with which participants had encountered REGAIN cases and the determinations they had made about patient enrollment. For each patient case, we asked the interviewee to open the record, then elicited an open-ended narrative about how the interviewee determined this patient was suitable or unsuitable for randomization. As this account developed, we posed scripted and spontaneous follow-up probes about how specific factors played into their reasoning: e.g., the patient’s medical history, the interviewee’s stance toward regional and general approaches, patient and family input, institutional standards, and the interviewee’s views toward the REGAIN trial. Once all patient cases had been discussed and the chart-stimulated portion was complete, we carried out a conventional semi-structured interview. In this section, we asked participants about their general views on clinical research, the role of physicians in facilitating clinical research, and any barriers that they felt hindered their participation in clinical studies.

### Analysis

Interviews were transcribed by a professional service. We used the NVivo qualitative analysis software (QSR International) to manage the coding. We began analysis while data collection was still underway, allowing us to determine when sufficient interviews had been collected to ensure theoretical saturation—i.e., the point at which the addition of new data did not alter the explanation we were developing [39].

To begin the coding process, each author independently reviewed a subset of 3 transcripts to identify themes. We then met as a team to discuss these themes and formalize them into a codebook—a taxonomy for categorizing qualitative data. Using this codebook, two authors (CD, MH) independently coded the same subset of 6 transcripts and met regularly to compare the coding, discuss the discrepancies, and refine the codebook to rectify ambiguous codes, eliminate redundant codes, and increase the comprehensiveness of the codebook. Having developed a refined codebook and agreement on its use, CD and MH then divided up and single-coded the remaining transcripts. Once this initial coding was complete, CD and MH performed focused coding [35] to identify the codes most pertinent to our research questions and posit potential connections between relevant codes. CD and MH were supervised during the coding process by JC and MN (an anesthesiologist, health services researcher, and PI of the REGAIN trial).

Having finished coding, we turned to explanation development. JC and MN undertook explanation development in an abductive process [40, 41] informed by prior literature on relevant topics as well as by our respective backgrounds as a social scientist and a trialist, respectively. We posed explanations for apparent trends, inductively examined these potential explanations to assess their degree of support from our interview data, and by doing so iteratively revised them until we arrived at a theory best supported by our findings. JC and MN met regularly as part of this iterative process.

## Results

We invited 62 anesthesiologists to participate, of whom 24 did not respond, 5 declined, 3 responded affirmatively but did not respond to subsequent scheduling requests, and 30 (48%) agreed and were interviewed. The interviews were conducted from August 2020 to June 2021. One interview was not completed because the interviewee experienced an interruption; this interview was excluded from the study. The mean interview length was 52 min. The characteristics of the 29 participating physicians are reported in Table 1. The characteristics of the 5 institutions where interviewees were practicing during the REGAIN trial are displayed in Table 2. We describe our interview findings below.

**Table 1:** Interviewee characteristics

**Characteristic**	**No. (%)**
Role in REGAIN	
Site PI	4 (14)
Sub-investigator	2 (7)
Site clinician	23 (79)
Years in practice	
0–10	13 (45)
11–20	10 (34)
21–30	2 (7)
31–40	4 (14)
Hip fracture cases per year (self-reported)	
0–10	10 (34)
11–30	8 (28)
31 +	11 (38)

**Table 2:** Characteristics of REGAIN sites where interviewees practiced during the trial

**Hospital ID**	**Clinicians interviewed**	**Country**	**Ownership**	**University affiliation**	**Top 10 enroller**
1	11	Canada	Public	Y	Y
2	11	USA	Private	Y	Y
3	3	USA	Private	N	N
4	2	USA	Public	Y	N
5	2	USA	Public	Y	N

### The anesthesiologist’s role: controlling complications

Physician interviewees described their approach to patients eligible for REGAIN as being typical of any hip fracture case. Their first step was scanning the medical record for any features that might present complications for spinal or general anesthesia. High sensitivity to contraindications was regarded as perhaps the central trait of the good anesthesiologist, as interviewees viewed spotting and adjusting to indicators of future trouble as their primary peri- and intraoperative tasks. “Nobody comes to hospital for an anesthetic,” stated one interviewee. “They come for other things. So that’s why our job is to mitigate risks, all the time.” (interviewee 10). The choice between spinal and general anesthetic was perceived as one of the anesthesiologist’s main sources of control over a patient’s trajectory.

As anesthesiologists, we like to have control. And so when some things are more risky, when some things are more unpredictable, we like to control all the things that we can. […] The drugs you do, the type of anesthetic you provide, these are things that you can definitely control. (interviewee 3)

The anesthesiologists we interviewed were concerned mainly with short-term threats to patient safety: for example, issues with the delivery of the anesthetic, with completing the operation, or with how the patient recovered in the immediate post-operative period. As one interviewee put it:

[W]hen you see that a patient’s journey from the admission to discharge is a line—like, we [anesthesiologists] intersect at a particular phase. So that patient actually has to travel the rest of the pathway […]. And I always feel that being an anesthetist, we actually intersect the journey for a very short duration of time. Of course, it does matter to them about what we do. […] But I don’t see or don’t decide what will happen three days postop. Do you know what I mean? (interviewee 25)

### Problematic cases I: Prototype-based reasoning

In the cases we discussed with interviewees, the single patient characteristic they most often flagged as a potential source of serious complications was any indication of dementia. Patients with dementia were of concern to anesthesiologists particularly when it came to the provision of spinal anesthesia, due to the possibility that they would not remain still and cooperative during the administration of the anesthetic or during the operation. In evaluating whether they were willing to give a spinal anesthetic to a given patient with dementia, anesthesiologists compared the case to the prototypical characteristics of what they called the “pleasantly demented” versus “non-pleasantly demented” patient. These characteristics were based on anesthesiologists’ prior clinical experience. Whether and how they manifested in the cases of specific patients eligible for REGAIN was inferred from behavioral signs picked up during interaction with these patients. For example, the below interviewee discusses a patient whom they withdrew from the trial due to a lack of willingness to administer spinal anesthesia.

He was a non-pleasantly demented 96-year-old. […] When he was randomized initially, he was in his bed, in his room with a family member, and he was nice and still and pleasant. Once you got him out of that environment, he became agitated to the point that I thought it might not be safe for the patient and to risk the surgical team to have just a spinal. […] It didn’t become clear until he came to the [operating room] environment that the original randomization might not have been suitable for him. But before that moment, I think I would have been entirely comfortable randomizing the patient. (interviewee 4)

“There are little clues,” said another interviewee about patients with dementia.

Things like sometimes patients will arrive in restraints, and that tells you they’re vigorous enough to be a danger to themselves but disorganized enough to need to be restrained. Right? […] Other [times] patients are hyperactive, who just move around the whole time or are completely unable to cooperate, there’s no eye contact, there’s no verbal response. It’s very much just from experience knowing which ones cope and which ones don’t. (interviewee 18)

Interviewees’ comfort performing the spinal anesthesia procedure in patients with dementia factored into how they considered which class a given patient represented. Said one anesthesiologist (interviewee 21) when recounting a patient with dementia whom he refused to randomization: “I mean, I have tried to do spinal anesthetics or epidurals in patients who have limited cognitive abilities and my success rate personally in doing these is usually very low.”

Hesitation about how to proceed with randomization in these cases centered on indeterminacy about how they compared to the prototypic situations in which anesthesiologists thought spinal tended to go well or poorly in patients with dementia. Once the patient was aligned with one or the other prototype, this discomfort was resolved, as there were clear implications for care. For example, the interviewee above (interviewee 4) who had determined a patient was “non-pleasantly demented” and withdrew him from randomization explained, “I thought this guy was probably in that category. So I thought, well, I won’t even try to give you a spinal. You’re just going to sleep.” “Had he been pleasantly demented,” said the same interviewee, “he would have had a spinal, no question in my mind.” “That sort of patient,” another anesthesiologist said of an individual with dementia they refused to randomize, “I mean, you can’t even consider doing it.” (interviewee 18).

### Problematic cases II: Probabilistic reasoning

The prototype-based reasoning that anesthesiologists used to make decisions about randomization in cases involving dementia contrasted with the probabilistic judgments they made in other scenarios. One situation in which interviewees consistently reasoned probabilistically was when caring for patients with multiple sclerosis, another contraindication they commonly highlighted in REGAIN cases. They worried that spinal anesthesia could cause a relapse in the condition. Below are two examples of interviewees describing patients they refused to randomize due to concerns over multiple sclerosis.

[I]t’s been known for some time that the combination of anesthesia, both general and spinal, and surgery can actually trigger a relapse of [multiple sclerosis]. Now, the problem with that, it’s difficult to tease out how much the surgery versus the anesthesia contributes to that possibility. […] But we also know that a spinal anesthetic is more likely to cause that versus a general anesthetic. […] Now, the evidence is not so clear-cut. The problem is you could always find somebody that would fight her corner [in] court. […] I might not have lost the suit, but nobody wants to go to court, right? […] When she came to the OR, I thought long and hard about how to do this. So it wasn’t like, oh yeah, that’s it. It wasn’t black and white. For her, it was a gray area. (interviewee 4)

So, she was allocated to the spinal anesthetic, but she had a history of multiple sclerosis. […] [B]ecause it’s essentially a demyelinating disease, there’s always the tendency not to undertake spinal anesthesia in case it can either cause the occurrence of the demyelination or cause progression of the disease. Although, the stress of the surgery may be associated with disease progression or reoccurrence. Itself, it might not be the spinal anesthetic […]. So there’s a tendency to avoid doing spinal anesthetics in people with multiple sclerosis. […] It could be the contributor, or it could confound it. (interviewee 22)

Several features of these accounts are notable. First, the anesthesiologists attribute their various propositions about multiple sclerosis to the professional collective rather than to their individual experience (e.g., “we also know,” “there’s a tendency to avoid doing spinal”). Second, they at least imply that they are drawing on research data (e.g., “the evidence is not so clear-cut”), and they speak in the language of statistical relationships (e.g., “may be associated with disease progression,” “could be the contributor, or it could confound it”). Third, their hesitation in determining whether to randomize does not stem from trying to ascertain what type of patient they have encountered—these patients are simply accepted as having a multiple sclerosis diagnosis—but rather from the indeterminacy of how the body will react to spinal intrusion and thus whether a good or bad outcome will occur.

### Accepting randomization: confidence and comfort

When interviewees discussed patients whose randomization to REGAIN they accepted, they described feeling confident that either anesthesia approach would be safe for these patients.

I would’ve done either one. I’m comfortable doing either technique. […] The patient was within the safety factors for both techniques. […] I kept it within the realm of safe care for the patient. The techniques had to follow safe medical practice for me to be even considering REGAIN for this to pick techniques randomly. (interviewee 12)

I thought that he would have done fine with either anesthetic. […] This patient didn’t have significant heart disease. While he was billed as someone with COPD and well-controlled asthma, his pulmonary status was fine. So I also thought a general anesthetic would have been just fine for him, as well. (interviewee 6)

In these cases, anesthesiologists’ determination to proceed with randomization usually did not rely on drawing an equivalence between the outcomes that would be achieved by the respective anesthesia approaches. Rather, the approaches were deemed to independently meet the anesthesiologist’s standard for what constituted safe care. In a minority of randomized cases, interviewees did compare the options, as in the following:

[The patient was] super sharp. He was coherent. […] And it was a very easy decision. […] I mean, if he wasn’t a REGAIN patient, given his mental status was so good and his age, I would have probably tried to tell him that I thought spinal was safer. But I didn’t think there was a huge differential, so we went ahead and randomized. […] Sometimes I had to speak to family members and say, I wouldn’t even be approaching you if I didn’t believe that either way was safe for your family member. (interviewee 7)

In rare accounts such as this, however—in which a comparison of the relative benefits of the approaches appears to have driven the determination to OK randomization—the disposition evinced remained one of confidence that both options would ultimately allow for a safe operation.

### Fiduciary relationship with patients and families

When it came to discussing their reasoning with patients and families, interviewees described a fiduciary commitment to communicate any concerns they had about a given anesthetic option. Reflecting on whether to offer recommendations about the anesthetic approach to patients with hip fractures or their surrogates, one anesthesiologist drew the following analogy:

[I]f I go in to meet with my financial advisor, and he says, “Well, you know what? You have X amount of dollars here. Here are the five things you can do with the money, and I won’t give you my personal opinion at all on what is the best.” I might be sitting there and saying, “Well, why did I actually come to you? You’re the specialist. What am I talking to you for […]?” So that’s how I see it for patients, too. (interviewee 9)

Interviewees felt that patients and families held similar expectations and recounted being asked to present their opinion on which anesthetic was preferable even after patients or proxies had agreed to enroll in REGAIN when approached by study staff.

Patients will sometimes come down consented, but then they’ll very much want to know what my personal feeling is one way or the other. […] [I]f they ask me for my opinion, what would I do if I was treating my parent, I’ll usually present my opinion for that and then that will actually affect their decision. (interviewee 21)

Whether done by their own initiative or in response to patient or family prompting, most interviewees saw conveying a recommendation as necessitated by their fiduciary duty even if it created problems for REGAIN enrollment. A few added that they felt being as transparent as possible about their concerns was a means of empowering the patient to make the most informed choice about whether to participate in the trial. For example, said one anesthesiologist of two patients he withdrew from the trial:

[I]n the two patients I discussed, the ones with the COPD [chronic obstructive pulmonary disease] and the MS [multiple sclerosis], I think when they’re enrolled into the study, there’s not a lot of discussion given to them. […] They didn’t have any frank discussion [with research staff] about the MS or the COPD, and then the implications of that. So, I think we shouldn’t be obstructive, but I think it’s important that the patients are fully informed, so that they can make the choices. (interviewee 22)

### Support for clinical research: perceived “barriers” to participation

When interviewees were asked for their general attitudes toward research, they universally proclaimed strong support. Research is “a necessity for the advancement of medicine,” one anesthesiologist said (interviewee 23), is “absolutely necessary to improve care,” responded another (interviewee 15), and is “the only way we’re gonna move forward,” said a third (interviewee 14). Such categorical statements were common, as were avowals that clinical research is a major influence on how they practice. “I’ve always based my practice […] on the best-practice models from current research,” remarked one anesthesiologist (interviewee 12). This enthusiasm extended to the REGAIN trial, which many interviewees stressed would usefully inform how they approach hip fracture cases.

If the REGAIN study shows me that there’s ten percent better outcomes with people who get a spinal, I’m gonna try to put a spinal on every single hip fracture patient, because we’d be providing better care. (interviewee 3)

Nearly all interviewees said that participation in clinical research should be viewed as a responsibility for physicians. Two interviewees opined that the rights of physicians who chose not to participate in research should be respected. One said that not all physicians should participate in research, basing this view on a concern that unenthusiastic physicians will not rigorously follow study protocols.

When we asked anesthesiologists what in their experience were general “barriers” to integrating clinical research into their practice, their responses focused on production pressure and associated lack of time, the potential for clinical studies to disrupt workflow (e.g., having to answer the “stupid phone calls from the research assistants” (interviewee 19)), and the failure to sometimes get timely notifications about the randomization of their patients from study staff. They did not bring up discomfort with patient eligibility determinations as a “barrier,” despite having often talked extensively about this issue in the chart-stimulated portion of the interview a few minutes earlier.

## Discussion

In this study, we sought to get a detailed sense of how practicing clinicians reason about whether to proceed with or refuse randomization of patients eligible for pragmatic trials. We interviewed anesthesiologists participating in REGAIN, a pragmatic trial on anesthesia in hip fracture. We found that, in line with their perceived role as preventors of any peri- and intraoperative complications, anesthesiologists approached the cases of eligible patients by focusing intently on contraindications to the delivery of one or the other anesthetic being tested. Interviewees sometimes reasoned about eligible patients using a prototype-based approach in which their concern was whether a patient was representative of a class of problematic patients. In other cases, their reasoning was probabilistic, focused on whether a given type of patient would experience complications from a particular anesthetic. In contrast to the feelings of discomfort they expressed when they excluded patients from randomization, when anesthesiologists allowed randomization to proceed, they felt confident that either of the approaches being tested would be safe. Anesthesiologists saw themselves as having a fiduciary duty to patients and did not hesitate to provide recommendations, even when they recognized that doing so would complicate trial recruitment. Nevertheless, they voiced strong support for clinical research and participation in it, stating that their involvement was mainly hindered by production pressure and workflow disruptions.

The main strength of this study is its use of chart-stimulated interviewing to elicit detailed accounts of specific trial-eligible patients from the physicians who treated them. Prior studies that have examined why particular patients have or have not been enrolled in trials have examined informed consent conversations [27–32]. These studies are valuable for revealing communication problems, but they do not directly address the thought process behind a clinician’s approach to presenting the trial to a patient. Researchers have also surveyed and interviewed clinicians about their general views on participation in trials, turning up findings similar to what we found when we asked about such views: high enthusiasm for research [42, 43] and an emphasis on time, workload, and workflow logistics as hindering participation [42, 44–48]. In a few studies, clinicians expressed general discomfort with a trial’s eligibility criteria in interviews [21, 27, 49]. As our findings demonstrate, such statements do not necessarily align with how clinicians reason in specific cases, nor do they reveal the complexities of this reasoning.

Our findings have several implications for attempts to evaluate and intervene in physicians’ tendency to exclude patients from clinical trials. First, though equipoise remains the most prominent conceptual tool for examining the ethical basis for physician determinations about study enrollment, it has questionable utility for addressing the reasoning of the anesthesiologists who participated in REGAIN. The traditional definition of equipoise grounds it in a particular metacognitive state, namely a feeling of “genuine uncertainty regarding the comparative merits of treatments A and B for population P” [10]. In such a state, a physician is typically deemed ethically justified in refusing to randomize a patient. Conversely, when a physician “knows that these treatments are not equivalent, ethics requires that the superior treatment be recommended” [10]. In the abundant literature on equipoise, its equivalence with uncertainty tends to imply that the physician willing to randomize a patient admits a lack of knowledge about which treatment is superior, while the physician who believes they know what is best has confidence even in the face of scant evidence. Our interviews complicate these assumptions. It was when anesthesiologists *refused* to randomize eligible patients that they were hesitant and uncomfortable. (Is this patient with dementia the type that can cope or the type that cannot cope with spinal anesthesia? What is the likelihood that something bad will happen to this patient with multiple sclerosis if a spinal anesthetic is administered?) Whereas when clinicians went ahead with randomization, they usually did not even directly compare the anesthetic options, instead expressing confidence that both would be safe—that the success of the operation would not be jeopardized by the trial. This suggests that refusal to randomize patients is not always the result of clinicians’ (over)confidence in their own reasoning. Interventions to facilitate trial enrollment may need to shore up physician anxieties about how participation could be detrimental to their ability to meet the standards of their specialty as much as they need to correct physician biases.

Our results also complicate arguments made to spur on the development of the “learning health system.” [16–18] Bioethicists and health policy experts have argued that a lowering of the threshold for meeting equipoise is both justified and will allow for easier integration of trials into clinical practice. These authors draw a tight equivalence between medical practice and research. They maintain that when there is “little empirical evidence” to support a clinician’s judgment that one therapy is superior, “[t]he obligation to respect clinician judgment in this context is not as stringent as in a case where clinician judgment is based on more robust evidence,” [17] and thus the clinician should permit randomization of the patient. This argument assumes clinical reasoning is always or predominantly probabilistic and thus that reference to research data is the sole or primary way in which clinicians evaluate treatment options. As exemplified by our interviewees’ judgments about patients with dementia, clinicians often draw on know-how derived from experience with similar situations. The objective of such reasoning is to establish a gestalt grasp of the situation through analogy with prototypic characteristics generated from prior cases they have encountered [4, 50]. Since this kind of reasoning is highly dynamic and context-sensitive, understanding how and in what situations it plays out, and how it will interact with trial eligibility criteria, requires close examination of clinical practice. Prototype-based reasoning is evidently difficult for clinicians to comment on in the abstract, given that in our interviews it only surfaced in the chart-stimulated portions. It was never, for example, reflected on as a “barrier” to interviewees’ participation in clinical research, and when interviewees expressed their general support for REGAIN, some even made statements in which they themselves seemed to presume that all of their reasoning was probabilistic and data-driven.

Finally, the differing modes of clinical reasoning involve different types of uncertainty. Uncertainty is not a monolithic phenomenon; it has varying sources and manifestations [51]. When clinicians excluded patients from randomization as a result of prototype-based reasoning, they were presented with ambiguity about what type of patient was in front of them, as they tried to synthesize a variety of co-occurring, potentially conflicting behavioral signs. However, once the patient was aligned with a prototype, the ambiguity was largely resolved. There was a deterministic relationship between, for example, the “non-pleasantly demented patient” and the problems that the attributes of such a patient would cause during and after the administration of spinal anesthesia [52]. In contrast, when clinicians excluded patients based on probabilistic reasoning, they faced indeterminacy about what would happen if a given anesthetic was used for this type of patient. Their reasoning was ostensibly based on outcomes data, and the inconclusive nature of this data generated the indeterminacy. It is important to differentiate the types of uncertainty that create problems for trial enrollment. They suggest distinct approaches to assessing whether clinical judgments are unwarranted or are instances of the kind of local adaptation that is necessary to make any standard protocol function [53, 54]. They also likely demand different approaches for effective intervention, if it is deemed necessary. For example, in the case of eligible patients with dementia who were excluded from the trial, a focus on the assumptions underlying anesthesiologists’ assignment of patients to the class of “non-pleasantly demented,” on the role of their procedural comfort administering spinal anesthesia in these categorizations, and on communication between the anesthesiologist and surgical team about such patients might be most productive. In the case of multiple sclerosis, a focus on anesthesiologists’ familiarity with and interpretation of outcomes data might be best.

### Limitations

This study has important limitations. We carried out interviews, not direct observations of clinical practice. These interviews are retrospective, rationalized accounts; missing are the aspects of real-time practice that are not so easy to reflect on and articulate. Relatedly, it is possible that interviewees’ recall was sometimes faulty. We tried to mitigate both these effects by drawing on the concreteness of real cases explored through chart stimulation and careful probing. The rich, multidimensional nature of the accounts we obtained suggests that this was to some degree successful. Interviewees’ accounts might also reflect social desirability bias. We tried to blunt this tendency by having non-clinicians who did not have leading roles in REGAIN and had not previously corresponded with participants conduct all interviews and all recruitment communications. The study also has characteristics that likely limit its generalizability. Our sample of interviewees was derived mainly from two high-enrolling sites that presented abundant cases to discuss. It is possible that physicians at sites with lower enrollment dealt with different circumstances when reasoning about patient cases, reflecting differences in clinical and research infrastructures across sites. Our findings are also specific to one pragmatic trial in one medical specialty with its own norms and practice patterns. However, we believe the findings of this study stress the importance of examining the specifics of a given trial’s clinical setting for evaluating and effectively intervening in clinicians’ tendency to refuse enrollment of eligible patients.

## Conclusions

This study lays out several features of clinical reasoning involved in whether physicians enroll patients in randomized trials. Prior research has demonstrated the importance of a well-rounded approach to assessing recruitment problems in the pilot or main phase of trials [25, 26]. As pragmatic trials become increasingly common, given that they study widely used treatments in settings of quotidian practice, it will be important to supplement this approach with an understanding of how clinicians typically reason about the procedures being tested. Crucially, this work can be done before a trial starts to help anticipate the specific scenarios that will often lead to patient exclusion based on clinical discretion. Having identified these specific scenarios, efforts such as focused training for participating clinicians aimed at increasing facility and comfort with such cases may be undertaken. This kind of tailored, clinician-level intervention might be sufficient to address certain problematic scenarios. Yet, in more extreme cases, scenarios may be so problematic that they make strict randomization infeasible. In such cases, alternatives to traditional randomized trial designs, or non-randomized studies, may be the best options. Finally, we conclude by stressing that more work is necessary to adequately assess the ethics of clinician refusal to randomize. The literature on this topic sometimes casts aspersions on clinicians who do not enroll eligible patients in trials by framing this activity as violating patient autonomy or impeding medical progress [55–57]; more often, it approaches this behavior as simply something to be overcome. As our findings reveal, such judgments are often grounded in questionable assumptions about how clinicians come to their conclusions about trial enrollment.

## Supplementary Information

**Additional file 1.** Interview guide.

## Data Availability

The datasets generated and/or analyzed during the current study are not publicly available to ensure the anonymity of the interviewees is preserved.
