# Supplementary material for: Clinical reasoning in pragmatic trial randomization: a qualitative interview study
Source: Trials. 2023 Jun 27;24:431. doi: 10.1186/s13063-023-07445-3 (PMC10294416; doi:10.1186/s13063-023-07445-3)
Supplement: Supplementary file 1 — Additional file 1. Interview guide. [file 13063_2023_7445_MOESM1_ESM.docx]

**SUPPLEMENTAL MATERIALS**

**Interview guide**

I’d like to start off by focusing generally on the REGAIN trial.

1. Which of the following descriptions best characterizes your role in the REGAIN trial at your site? (1) Site lead or co-lead investigator; (2) Site sub-investigator; (3) Other clinical team member.
2. On a scale of 1-5, with 1 being “not at all familiar” and 5 being “extremely familiar,” how would you rate your familiarity with the REGAIN trial?
3. In your own words, what are the basic goals of the REGAIN trial?
4. In your own words, what makes a patient eligible for REGAIN?
5. Chart-stimulated

[FOR EACH CASE]

Now, I’d like to talk with you about a specific patient you treated who was eligible for or enrolled in REGAIN.

Can you please confirm that you have access to the medical record now? Could you please pull up the anesthesia record for [PID]?

1. On a scale of 1-5, with 1 being no recall and 5 being perfect recall, how would you rate your recall of this case?
2. What was your role in taking care of this patient? Do you recall having to make a decision as to whether the patient would be a suitable candidate for randomization to spinal versus general anesthesia through participation in REGAIN?

I’d like to focus in now on the decision as to whether the patient would be suitable for randomization to either spinal or general anesthesia through participation in REGAIN. If you recall personally making this decision, please comment based on your recollection of the case. If you don’t recall this or weren’t involved, please comment on what you most likely would have done if this case was presented to you as a potential REGAIN patient.

1. Can you tell me about how you determined whether the patient was a suitable candidate for randomization?
   1. How did aspects of the patient’s medical history factor into this decision?
      1. Looking at the chart now, are there other aspects of the patient’s medical history aside from ________ that you think may have affected your decision?
   2. Do you have a usual preference for spinal or general anesthesia? How did factor into this decision?
   3. How did patient and family preferences factor into this decision?
   4. How did institutional standards of care factor into this decision?
   5. How did your comfort with the general practice of randomizing, factor into this decision?
2. To what extent did maintaining fidelity to the eligibility criteria for REGAIN factor into your decision?
3. Overall, how easy or difficult was it for you to make this decision to not/randomize the patient?
   1. IF WITHDRAWN: Of the factors you previously mentioned, what would have to be different for you to reconsider withdrawing this patient?
4. If REGAIN did not exist, what are some ways your decision-making process would have been different from what you previously described, if at all?
5. General semi-structured

For the final portion of the interview, I’d like to ask you a few general questions about your practice and your general thoughts on clinical research studies.

1. For how many years have you been practicing independently?
2. Over the last 12 months, about how many hip fracture cases have you provided care for?

Now I’d like to get your thoughts on RCTs generally.

Please indicate your level of agreement with the following statements on a scale of 1-5, where 1 corresponds to “disagree completely,” and 5 corresponds to “agree completely:”

1. “I have a positive view about medical research in general.” (RAQ q1)
   1. Can you explain the reasons why you’ve given this number?
2. “Clinicians all have some responsibility to help others by facilitating medical research.” (RAQ q4)
   1. Can you explain the reasons why you’ve given this number?

[IF RESPONSE IS 3-5]

- - 1. What are some ways clinicians can or should facilitate medical research?
    2. How should clinicians weigh what they think is right for a particular patient against the ability for a case to contribute to research?

[IF RESPONSE IS 1-2]

- - 1. Should clinicians have any responsibility to facilitate to medical research? If so, what should these responsibilities be? If not, what role should clinicians play in research, if at all?
    2. How should clinicians weigh what they think is right for a particular patient against the ability for a case to contribute to research?

1. What general barriers make it difficult to integrate clinical research into your typical clinical practice?
2. Is there anything I didn’t ask about that you think is important in making decisions about enrolling your patients in randomized studies?

Thank you very much for your time. This concludes the interview.
